# Family obligation and Chinese adolescents’ wellbeing: the moderating role of school connectedness

**DOI:** 10.3389/fpsyg.2026.1888336

**Published:** 2026-08-05

**Authors:** Yuan Liu

**Affiliations:** 1 Department of Student Affairs, Weinan Normal University, Weinan, China; 2 School of Teacher Development, Shaanxi Normal University, Xi’an, China

**Keywords:** Chinese adolescents, eudaimonic wellbeing, family obligation, life satisfaction, school connectedness

## Abstract

Wellbeing is essential for healthy adolescent development, yet the association between family obligation and adolescents’ wellbeing remains underexplored, particularly within collectivistic cultural contexts. The present study examined the associations between family obligation and Chinese adolescents’ eudaimonic wellbeing and life satisfaction, as well as the moderating role of school connectedness in these associations. Participants were 2,259 secondary school students (mean age = 15.55 years) from three Chinese provinces (Shaanxi, Xinjiang, and Jilin), who completed an online survey assessing family obligation, school connectedness, eudaimonic wellbeing, and life satisfaction. Observed-variable path analyses within the structural equation modeling (SEM) framework and simple slope analyses were conducted. The results showed that family obligation was positively associated with both eudaimonic wellbeing and life satisfaction among Chinese adolescents. Furthermore, school connectedness significantly moderated these associations, such that the positive associations between family obligation and both dimensions of wellbeing were stronger among adolescents with higher levels of school connectedness. These findings highlight the joint associations of family and school microsystems with adolescents’ wellbeing and contribute to a better understanding of adolescent wellbeing within the Chinese collectivistic cultural context. They also provide empirical evidence that may inform future family- and school-based efforts to support adolescent wellbeing.

## 1 Introduction

Adolescence is a critical developmental period during which individuals undergo substantial biological, cognitive, and social transitions, making the promotion of wellbeing a central developmental task (Bücker et al., 2018; Qu et al., 2025; Zhang et al., 2021). Among the various family-related factors associated with adolescent wellbeing, family obligation has attracted growing scholarly attention because it reflects adolescents’ willingness to support, respect, and reciprocate their family members (Fuligni et al., 1999; Fuligni and Pedersen, 2002). This construct is particularly salient in collectivistic societies such as China, where family interdependence, filial values, and family responsibilities are strongly emphasized throughout adolescence (Yang et al., 2024).

Despite accumulating evidence linking family obligation to positive developmental outcomes, its implications for adolescent wellbeing remain theoretically ambiguous. On the one hand, fulfilling family responsibilities may be associated with a stronger sense of belonging, competence, and self-worth, which may in turn be related to more positive psychological functioning (Juang and Cookston, 2009; King and Ganotice, 2015; Oh et al., 2020; Telzer and Fuligni, 2009; Yan et al., 2022; Yang et al., 2024). On the other hand, higher levels of family obligation may be associated with greater psychological burden and role conflict, particularly when adolescents experience competing developmental demands from school, peers, and family (Telzer et al., 2015; Wilkinson-Lee et al., 2011). These seemingly inconsistent findings suggest that family obligation is neither inherently beneficial nor detrimental. Instead, its developmental implications may depend on the broader ecological contexts in which adolescents experience and interpret their family responsibilities.

Consequently, a more theoretically meaningful question is no longer whether family obligation is associated with adolescent wellbeing, but rather when and under what contextual conditions family obligation is associated with more positive wellbeing. Addressing this question is particularly important in the Chinese cultural context, where family obligation constitutes a normative developmental expectation and may interact with adolescents’ experiences in other important developmental settings, especially school. Identifying the contextual conditions under which family obligation is associated with positive functioning, therefore, represents an important step toward advancing theoretical understanding of adolescent wellbeing.

### 1.1 Family obligation as a culturally embedded developmental resource

Family obligation refers to adolescents’ beliefs, attitudes, and behaviors reflecting responsibility, assistance, and reciprocity toward family members (Fuligni et al., 1999; Fuligni and Pedersen, 2002). Although family obligation has been widely studied across different cultural contexts, its developmental meaning varies substantially across societies. In individualistic cultures, family obligation is often viewed as an individual choice or personal value, whereas in collectivistic societies such as China, it represents a culturally endorsed developmental norm rooted in filial piety, family interdependence, and collective responsibility (Yang et al., 2024). Consequently, fulfilling family obligations is not merely a behavioral expectation but also an important pathway through which Chinese adolescents construct their social identity and maintain harmonious family relationships.

Existing studies have consistently reported that family obligation functions as a double-edged developmental resource. Rather than producing uniformly positive or negative consequences, its influence depends on how adolescents experience and manage their family responsibilities.

On the positive side, family obligation provides adolescents with opportunities to develop responsibility, strengthen family cohesion, and receive emotional support from family members. Longitudinal evidence suggests that stronger family obligation was associated with fewer depressive symptoms by enhancing adolescents’ sense of belonging within the family (Juang and Cookston, 2009). Likewise, engaging in daily family assistance, an important behavioral manifestation of family obligation, has been associated with greater psychological wellbeing because helping family members enhances adolescents’ self-worth and social connectedness (Telzer and Fuligni, 2009). Among Chinese adolescents, positive family obligation attitudes and behaviors have also been linked to lower levels of negative emotions and healthier psychological adjustment, largely because fulfilling family responsibilities is culturally valued and reinforces positive family interactions (Yang et al., 2024).

Nevertheless, family obligation may also become a source of developmental stress. Adolescents often need to balance family responsibilities with academic demands, peer relationships, and increasing autonomy. When family obligations exceed adolescents’ coping resources or conflict with other developmental tasks, they may be associated with greater psychological burden rather than psychological benefits. Wilkinson-Lee et al. (2011), for example, found that excessive family obligation was associated with greater emotional distress, whereas Telzer et al. (2015) reported that high family demands were associated with greater internalizing symptoms when adolescents lacked sufficient coping resources. These findings suggest that family obligation should not be conceptualized simply as a protective family factor. Instead, whether it is associated with more favorable or less favorable developmental outcomes likely depends on the developmental contexts in which these family responsibilities are enacted.

Taken together, previous research has established that family obligation is consistently associated with adolescents’ psychological adjustment but has paid relatively little attention to the contextual conditions under which these associations may vary. Addressing this limitation requires moving beyond documenting direct associations and instead examining whether the association between family obligation and adolescent wellbeing differs across important developmental contexts.

### 1.2 Adolescents’ wellbeing and their relation with family obligation

Wellbeing is a multidimensional construct that is commonly conceptualized from two complementary perspectives: hedonic wellbeing and eudaimonic wellbeing (Qu et al., 2025; Ryan and Deci, 2001). Although these two perspectives are positively related, they emphasize different aspects of optimal psychological functioning and therefore may respond differently to adolescents’ family experiences.

Hedonic wellbeing is generally conceptualized as comprising life satisfaction together with positive and negative affect (Ryan and Deci, 2001). Because life satisfaction represents the cognitive evaluation component of hedonic wellbeing (Busseri and Sadava, 2011; Diener et al., 2018) and is highly correlated with affective components (Busseri, 2018), the present study focused specifically on life satisfaction as an indicator of adolescents’ cognitive evaluation of their wellbeing. Accordingly, throughout this study, life satisfaction is examined as a distinct outcome rather than as a measure of the broader hedonic wellbeing construct. By contrast, eudaimonic wellbeing emphasizes functioning well, referring to realizing one’s potential, experiencing personal growth, and developing a meaningful and purposeful life (Huta and Waterman, 2014). During adolescence, these two dimensions jointly capture important developmental outcomes because successful adaptation requires not only experiencing life satisfaction but also developing a coherent sense of purpose, competence, and identity (Qu et al., 2025; Zhang et al., 2021).

The distinction between these two forms of wellbeing is theoretically important when examining family obligation. Family obligation may be more strongly associated with eudaimonic wellbeing because fulfilling culturally valued family responsibilities enables adolescents to experience meaning, competence, and social contribution (Fuligni et al., 2002; Yang et al., 2024). At the same time, positive family interactions and emotional support associated with family obligation may also be associated with higher levels of life satisfaction by increasing positive emotional experiences (King and Ganotice, 2015; Telzer and Fuligni, 2009; Yan et al., 2022). However, previous studies have typically examined general psychological wellbeing without distinguishing whether family obligation is similarly associated with these two theoretically distinct dimensions. Consequently, it remains unclear whether family obligation is similarly associated with eudaimonic wellbeing and life satisfaction.

Addressing this issue contributes to a more comprehensive understanding of adolescent wellbeing. Rather than treating wellbeing as a unitary construct, distinguishing life satisfaction and eudaimonic wellbeing enables researchers to examine whether family obligation is more strongly associated with adolescents’ life satisfaction, with their personal growth and meaning in life, or with both dimensions simultaneously. Such differentiation provides a more nuanced understanding of the developmental significance of family obligation and establishes the basis for examining the contextual mechanisms through which these associations may vary across adolescents.

### 1.3 School connectedness as a contextual boundary condition

The preceding review suggests that family obligation cannot be regarded as uniformly beneficial or detrimental. Instead, whether fulfilling family responsibilities is associated with adolescents’ wellbeing appears to depend on the developmental contexts in which these responsibilities are experienced. Identifying such contextual conditions is essential for advancing theoretical understanding of family obligation because it helps explain why previous studies have reported both positive and negative developmental associations. Guided by Bronfenbrenner’s ecological systems theory (Bronfenbrenner, 1992), the present study proposes that school connectedness represents one important contextual condition that shapes the developmental implications of family obligation.

School connectedness refers to adolescents’ subjective sense of belonging, acceptance, and support within the school environment, including positive relationships with teachers and peers as well as feelings of being respected and valued by the school community (Goodenow, 1993; Peng and Patterson, 2025). It encompasses three core dimensions: the perception of being cared for and respected by school staff, positive and supportive peer relationships, and a sense of involvement and commitment to school activities (Furlong et al., 2011; McNeely et al., 2002; Peng and Patterson, 2025; Yu et al., 2011). Considerable evidence has reported that school connectedness is positively associated with a wide range of developmental outcomes, including psychological adjustment, academic engagement, resilience, and wellbeing (Allen et al., 2018; Raniti et al., 2022; Rose et al., 2024; Yuen and Wu, 2024). However, most existing studies have primarily conceptualized school connectedness as an independent protective factor that is directly associated with positive developmental outcomes. Comparatively less attention has been devoted to its role as a contextual condition that may moderate the associations between developmental resources originating from other ecological settings (e.g., family) and adolescent adjustment.

Bronfenbrenner’s ecological systems theory provides a useful framework for addressing this issue. The theory argues that adolescent development is shaped not only by experiences within individual microsystems, such as the family and school, but also by the interactions among these microsystems, commonly referred to as mesosystem processes (Bronfenbrenner, 1992). From this perspective, adolescents do not experience family influences and school influences independently. Rather, developmental outcomes emerge through the dynamic interplay between these proximal contexts. Consequently, the developmental consequences of family obligation should not be expected to remain constant across all adolescents; instead, they are likely to vary according to the quality of adolescents’ experiences within the school environment.

Building on this perspective, we argued that school connectedness functions as a contextual resource that may facilitate the association between family obligation and positive developmental outcomes. Family obligation provides adolescents with opportunities to develop responsibility, reciprocity, and commitment toward significant others (Fuligni et al., 1999; Fuligni and Pedersen, 2002). Nevertheless, these family-derived values and motivations may not automatically be associated with higher wellbeing. Adolescents also require supportive social environments in which these values can be recognized, reinforced, and integrated into their broader self-concept. High levels of school connectedness provide such an environment by fostering supportive teacher-student relationships, positive peer interactions, and a strong sense of belonging (Goodenow, 1993; Peng and Patterson, 2025). These experiences offer adolescents emotional security, social affirmation, and opportunities to experience competence beyond the family context, thereby reinforcing the positive psychological meaning associated with fulfilling family responsibilities. Under such conditions, family obligation is more likely to be interpreted as an opportunity for contribution, personal growth, and social connectedness rather than as an external demand or developmental burden.

In contrast, adolescents with low school connectedness may experience fewer contextual resources that help them positively interpret their family responsibilities. A lack of belonging at school may reduce opportunities to receive emotional support and positive feedback outside the family, making family obligations more likely to be experienced as stressors that compete with academic and social developmental tasks. Consequently, even adolescents with a strong sense of family obligation may experience fewer psychological benefits when they lack a supportive school environment. School connectedness therefore represents more than another positive correlate of wellbeing; it may shape the extent to which the developmental resources embedded in family obligation are associated with adolescents’ positive psychological functioning.

Emerging empirical evidence provides preliminary support for this proposition. Wilkinson-Lee et al. (2011) found that school connectedness buffered the adverse emotional consequences associated with excessive family obligation, suggesting that supportive school experiences may modify how adolescents respond to family responsibilities. Likewise, Allen et al. (2018) suggested that school connectedness may strengthen the effectiveness of family-related protective factors by providing adolescents with a stable and supportive developmental context. Nevertheless, previous studies have rarely examined whether school connectedness moderates the association between family obligation and different dimensions of adolescent wellbeing, particularly among Chinese adolescents whose family obligations are strongly embedded within collectivistic cultural values.

Based on ecological systems theory and the emerging evidence on cross-contextual developmental processes, we proposed that school connectedness serves as a contextual boundary condition that helps explain under what contextual conditions family obligation is more strongly associated with adolescent wellbeing. Specifically, we expected that the positive association between family obligation and both dimensions of wellbeing would be stronger among adolescents with higher levels of school connectedness.

### 1.4 The current study

Despite growing evidence linking family obligation to adolescent adjustment, three important theoretical gaps remain. First, previous research has predominantly focused on the association between family obligation and psychological wellbeing (Telzer and Fuligni, 2009; Yan et al., 2022), whereas considerably less attention has been devoted to identifying the contextual conditions under which these associations may vary. Second, although ecological systems theory emphasizes the interaction among developmental contexts, relatively few studies have explicitly examined how experiences within the school microsystem may shape the association between family obligation and adolescent wellbeing. Third, previous research has generally conceptualized wellbeing as a single outcome, leaving it unclear whether contextual influences operate similarly across the theoretically distinct dimensions of eudaimonic wellbeing and life satisfaction.

**Figure 1:**
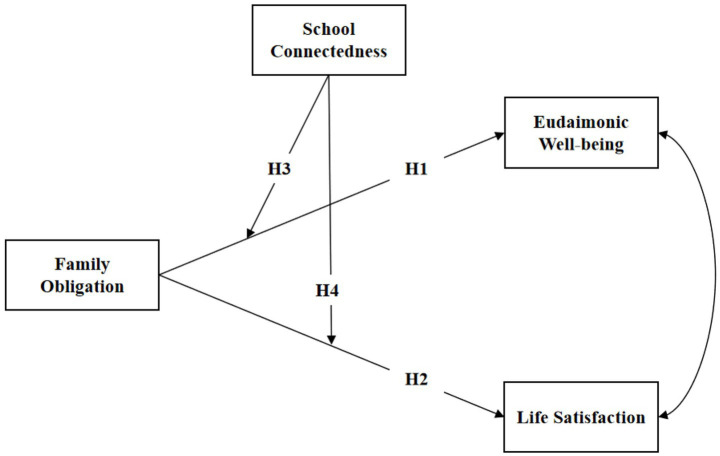
The conceptual model.

The present study addresses these gaps by examining school connectedness as a contextual boundary condition linking family obligation to adolescents’ wellbeing (see Figure 1). Guided by ecological systems theory (Bronfenbrenner, 1992), we conceptualized the interaction between family obligation and school connectedness as reflecting mesosystem processes through which family and school jointly relate to adolescent wellbeing. Furthermore, by simultaneously examining life satisfaction and eudaimonic wellbeing, the present study provides a more nuanced understanding of whether this cross-contextual interaction generalizes across different domains of positive functioning. Finally, focusing on Chinese adolescents offers an important opportunity to examine these processes within a cultural context where family obligation represents a particularly salient developmental value (Yang et al., 2024).

Accordingly, the present study contributes to the literature in three important ways. First, it shifts the theoretical focus from documenting the association between family obligation and adolescent wellbeing to examining the contextual conditions under which these associations are stronger, thereby identifying school connectedness as a contextual boundary condition. Second, it extends the application of ecological systems theory by emphasizing the interaction between family and school microsystems in understanding adolescent wellbeing rather than treating these developmental contexts as independent correlates. Third, by distinguishing life satisfaction and eudaimonic wellbeing, the study provides a more comprehensive understanding of whether family obligation is similarly associated with these two theoretically distinct dimensions of positive psychological functioning among Chinese adolescents.

Based on these theoretical considerations, we proposed the following hypotheses:

*H1*: Family obligation is positively associated with adolescents’ eudaimonic wellbeing.

*H2*: Family obligation is positively associated with adolescents’ life satisfaction.

*H3*: School connectedness positively moderates the association between family obligation and eudaimonic wellbeing, such that this positive relationship is stronger at higher levels of school connectedness.

*H4*: School connectedness positively moderates the association between family obligation and life satisfaction, such that this positive relationship is stronger at higher levels of school connectedness.

## 2 Methods

### 2.1 Participants

A total of 3,044 secondary school students, mainly from three Chinese provinces (Shaanxi, Xinjiang, and Jilin), were recruited through convenience sampling to participate in an online survey, which was distributed through a popular Chinese survey platform.^1^ To ensure data quality, all responses were subjected to a predefined data-screening procedure. First, 92 participants were excluded because they provided invariant responses (i.e., selecting the same response option for all questionnaire items), suggesting insufficient response engagement. Second, the survey included one attention-check item (e.g., “Please select ‘agree’ for this item”; Qu et al., 2024). Participants who did not select the required response option were excluded, resulting in the removal of 693 participants. After data screening, 2,259 participants were retained for the final analyses, representing 74.21% of the original sample.

The participants’ ages ranged from 11 to 19 years, with a mean age of 15.55 (SD = 1.64). In terms of provincial distribution, the sample included 1,188 (52.59%) participants from Shaanxi Province, 816 (36.12%) from Xinjiang Province, 209 (9.25%) from Jilin Province, and 46 (2.04%) from other provinces. Regarding school type, 1,521 (67.33%) students were recruited from urban schools and 738 (32.67%) from rural schools. Notably, this study primarily focused on individual-level psychological variables. Therefore, detailed school-level information (e.g., school identifiers, the total number of participating schools, school administrative type, and school ranking) was not systematically collected. Consequently, the distribution of participants across schools and potential school-level clustering could not be examined. Detailed information about the participants was presented in Table 1.

**Table 1:** Participants’ demographic characteristics (*N* = 2,259).

Variables	*n*	Percent
Gender		
(1) Male	985	43.60%
(2) Female	1,274	56.40%
Age		
(1) ≤15 years old	978	43.29%
(2) >15 years old	1,281	56.71%
Grade		
(1) Grade 7	244	10.80%
(2) Grade 8	295	13.06%
(3) Grade 9	162	7.17%
(4) Grade 10	727	32.18%
(5) Grade 11	421	18.64%
(6) Grade 12	410	18.15%
Province		
(1) Shaanxi	1,188	52.59%
(2) Xinjiang	816	36.12%
(3) Jilin	209	9.25%
(4) Other	46	2.04%
School type		
(1) Urban	1,521	67.33%
(2) Rural	738	32.67%
Whether the only child in family		
(1) Yes	464	20.54%
(2) No	1,795	79.46%

### 2.2 Procedures

The research protocol was reviewed and approved by the Ethics Review Board of the authors’ affiliated institution. Participants were recruited through collaborating teachers from secondary schools in the participating provinces. Prior to initiating data collection, online informed consent was obtained from all participants. The online questionnaire administered to participants included sections on demographic information, family obligation, school connectedness, eudaimonic wellbeing, and life satisfaction. Participation was entirely voluntary, and no monetary or material compensation was provided to participants. All participants were informed that they had the right to withdraw from the study at any point without any negative consequences.

### 2.3 Measures

#### 2.3.1 Demographic variables

Demographic information collected from participants included gender (1 = male; 2 = female), age, grade (1 = grade 7; 2 = grade 8; 3 = grade 9; 4 = grade 10; 5 = grade 11; 6 = grade 12), province (1 = Shaanxi; 2 = Xinjiang; 3 = Jilin; 4 = Other), school type (1 = urban, 2 = rural), and whether they were the only child in their family (1 = yes, 2 = no).

#### 2.3.2 Family obligation

The Attitudes toward Family Obligation Scale, developed by Fuligni et al. (1999) and further refined by Fuligni and Pedersen (2002), was used to assess participants’ family obligation. This scale comprises three dimensions: current assistance (11 items; e.g., “Spend time with your family on weekends”), respect for family (7 items; e.g., “Treat your parents with great respect”), and future support (6 items; e.g., “Help your parents financially in the future”). Items related to current assistance were rated on a 5-point Likert scale (1 = almost never to 5 = almost always), while items assessing respect for family and future support were scored from 1 (not important at all) to 5 (very important). The original English scale was translated into Chinese for use in this study. First, two doctoral researchers in educational psychology independently translated the original English version into Chinese. Subsequently, two professors in educational psychology who were proficient in both Chinese and English reviewed the translated version and provided suggestions for revision. Based on their feedback, the Chinese version was finalized and administered to all participants. The scale demonstrated excellent internal consistency (Cronbach’s *α* = 0.94). The three-factor structure model showed acceptable fit to the data (*χ*^2^(236) = 2,905.31, *p* < 0.001, CFI = 0.924, RMSEA = 0.071, SRMR = 0.047). The three first-order factors were moderately to strongly correlated (*r*s = 0.58–0.70, *p*s < 0.001).

Although current assistance, respect for family, and future support represent conceptually distinct facets of family obligation, they are theoretically conceptualized as complementary components of an overarching family obligation construct (Fuligni et al., 1999; Fuligni and Pedersen, 2002). Consistent with prior studies (Telzer et al., 2011, 2015), we therefore calculated a composite family obligation score by averaging all items, with higher scores indicating a stronger sense of family obligation. In addition, a second-order CFA model specifying a higher-order family obligation factor demonstrated identical model fit (*χ*^2^(236) = 2,905.31, *p* < 0.001, CFI = 0.924, RMSEA = 0.071, SRMR = 0.047), supporting the conceptualization of family obligation as a higher-order construct.

#### 2.3.3 Eudaimonic wellbeing

Consistent with previous research (Qu et al., 2025), the Flourishing Scale (Diener et al., 2010), which has been validated for use with the Chinese population (Tang et al., 2016), was employed to measure adolescents’ eudaimonic wellbeing. This scale consists of 8 items (e.g., “I lead a purposeful and meaningful life”), all of which were rated on a 7-point Likert scale (1 = totally disagree to 7 = totally agree). We averaged the eight items to represent eudaimonic wellbeing, with higher scores indicating a stronger sense of eudaimonic wellbeing. The scale exhibited good reliability in this study (Cronbach’s *α* = 0.95).

#### 2.3.4 Life satisfaction

Adolescents’ life satisfaction was assessed using the Satisfaction with Life Scale (Diener et al., 1985), which includes 5 items (e.g., “In most ways my life is close to my ideal”). Each item was rated on a 7-point Likert scale ranging from 1 (totally disagree) to 7 (totally agree). We averaged the five items to represent life satisfaction, with higher scores indicating a stronger sense of life satisfaction. The scale showed good internal consistency in the current research (Cronbach’s *α* = 0.94).

#### 2.3.5 School connectedness

Consistent with recent research (Sani et al., 2026), we used the Adolescent Health’s School Connectedness Scale (Furlong et al., 2011) to assess Chinese adolescents’ school connectedness. This scale contains 5 items (e.g., “I feel close to people at my school” and “I am happy to be at this school”), all rated on a 5-point Likert scale (1 = strongly disagree to 5 = strongly agree). A composite score for school connectedness was computed by averaging the items, with higher scores reflecting stronger school connectedness. The scale demonstrated good reliability in the present study (Cronbach’s *α* = 0.91).

### 2.4 Control variables

Demographic variables, including gender, grade, province, school type, and whether the participant was the only child in their family, were included as covariates to account for potential demographic confounding. Although both age and grade were initially considered, we ultimately controlled only for grade because age and grade were highly correlated (*r* = 0.92, *p* < 0.001), indicating substantial conceptual and statistical overlap. Furthermore, including both age and grade simultaneously resulted in variance inflation factors (VIFs) of 6.77, suggesting potential multicollinearity. We therefore retained grade as the control variable because it more directly reflects adolescents’ educational context, which is theoretically more relevant to school connectedness and school-based developmental experiences than chronological age.

### 2.5 Data analysis

The online survey platform enforced complete response submission, ensuring that all questionnaires were fully completed. Confirmatory Factor Analysis was conducted to test the three-factor structure model of family obligation. Model fit was evaluated using three standard indices: Comparative Fit Index (CFI), Root Mean Square Error of Approximation (RMSEA), and Standardized Root Mean Square Residual (SRMR). Following widely accepted criteria (Hu and Bentler, 1999), a good model fit was defined as CFI ≥ 0.95, RMSEA ≤ 0.06, and SRMR ≤ 0.08, while an acceptable model fit was indicated by CFI ≥ 0.90, RMSEA ≤ 0.10, and SRMR ≤ 0.10. Descriptive statistics and bivariate correlation analyses were performed using SPSS 29.0. Observed-variable path analysis within the Structural equation modeling (SEM) framework was conducted in Mplus 8.3 (Estimation Method = ML, Maximum Likelihood) to test the primary research hypotheses. During the moderation analysis, an observed product (interaction) term was created by multiplying the raw (uncentered and unstandardized) composite scores of family obligation and school connectedness. When a significant interaction effect was detected, simple slope analyses were conducted following Aiken and West (1991). Specifically, the conditional effects of family obligation were estimated at one standard deviation above and below the mean of school connectedness using unstandardized regression coefficients.

## 3 Results

### 3.1 Common method bias

To evaluate the potential impact of common method bias on the study results, Harman’s single-factor test was applied to all measurement items. The analysis revealed that the first unrotated factor explained 42.01% of the total variance, which is below the commonly recommended threshold of 50% (Podsakoff et al., 2024; Qu et al., 2026). To further evaluate the potential influence of common method bias, an unmeasured latent method factor was incorporated into the measurement model following the recommendations of Podsakoff et al. (2024). The inclusion of the method factor resulted in virtually identical model fit indices (CFI = 0.957 vs. 0.957; RMSEA = 0.067 vs. 0.067; SRMR = 0.034 vs. 0.033). These findings suggest that common method bias did not exert a substantial influence on the current study’s outcomes.

### 3.2 Descriptive statistics

Descriptive statistics and bivariate correlations for all study variables were summarized in Table 2. Correlation analyses indicated that both family obligation and school connectedness were positively correlated with eudaimonic wellbeing and life satisfaction (*r*s = 0.57–0.65, *p*s < 0.001). Additionally, family obligation was positively associated with school connectedness (*r* = 0.54, *p* < 0.001), and a strong positive correlation was observed between eudaimonic wellbeing and life satisfaction (*r* = 0.72, *p* < 0.001).

**Table 2:** Descriptive statistical results for the study variables (*N* = 2,259).

Variables	1	2	3	4	5	6	7	8	9	10
1. Gender	–									
2. Age	0.04	–								
3. Grade	0.08^***^	0.92^***^	–							
4. Province	0.04^*^	0.29^***^	0.29^***^	–						
5. School type	−0.02	−0.45^***^	−0.47^***^	−0.34^***^	–					
6. Only child	0.09^***^	0.04	0.03	−0.08^***^	0.03	–				
7. Family obligation	−0.11^***^	−0.08^***^	−0.07^**^	−0.04	0.02	0.09^***^	–			
8. School connectedness	−0.13^***^	−0.17^***^	−0.16^***^	−0.06^**^	0.10^***^	−0.01	0.54^***^	–		
9. Eudaimonic wellbeing	−0.18^***^	−0.11^***^	−0.10^***^	−0.09^***^	0.07^**^	−0.01	0.57^***^	0.64^***^	–	
10. Life satisfaction	−0.20^***^	−0.13^***^	−0.14^***^	−0.08^***^	0.06^**^	−0.03	0.58^***^	0.65^***^	0.72^***^	–
*M*	–	15.55	–	–	–	–	4.10	4.11	5.79	5.35
SD	–	1.64	–	–	–	–	0.62	0.86	1.19	1.46

“Only child” refers to “whether the only child in family.” *M*, means; SD, standard deviations. ^*^p < 0.05; ^**^p < 0.01; ^***^p < 0.001.

### 3.3 Associations between family obligation and wellbeing

Observed-variable path analysis within the SEM framework was conducted to examine the associations between family obligation and adolescents’ wellbeing. The model was just-identified (*df* = 0, CFI = 1.00, RMSEA = 0.00, SRMR = 0.00). After controlling for demographic variables (gender, grade, province, school type, and only-child status; see Table 3), family obligation was significantly and positively associated with both eudaimonic wellbeing (*β* = 0.56, *p* < 0.001) and life satisfaction (*β* = 0.56, *p* < 0.001). The comparable standardized associations suggest that family obligation may serve as a common developmental resource underlying both eudaimonic and life satisfaction.

**Table 3:** Standardized results of the associations between family obligation and wellbeing.

Predictors	Dependent variable							
	Eudaimonic wellbeing (*R*^2^ = 0.35)				Life satisfaction (*R*^2^ = 0.36)			
	*β*	SE	*p*	95% CI	*β*	SE	*p*	95% CI
1. Gender	−0.11	0.02	<0.001	[−0.14, −0.07]	−0.13	0.02	<0.001	[−0.16, −0.09]
2. Grade	−0.03	0.02	0.171	[−0.07, 0.01]	−0.08	0.02	<0.001	[−0.12, −0.04]
3. Province	−0.05	0.02	0.007	[−0.09, −0.01]	−0.04	0.02	0.050	[−0.07, 0.00]
4. School type	0.03	0.02	0.174	[−0.01, 0.07]	−0.00	0.02	0.901	[−0.04, 0.04]
5. Only child	−0.05	0.02	0.002	[−0.09, −0.02]	−0.07	0.02	<0.001	[−0.10, −0.03]
6. Family obligation	0.56	0.01	<0.001	[0.53, 0.59]	0.56	0.01	<0.001	[0.53, 0.59]

“Only child” refers to “whether the only child in family.” SE, standard error; CI, confidence interval.

### 3.4 School connectedness as a moderator

**Figure 2:**
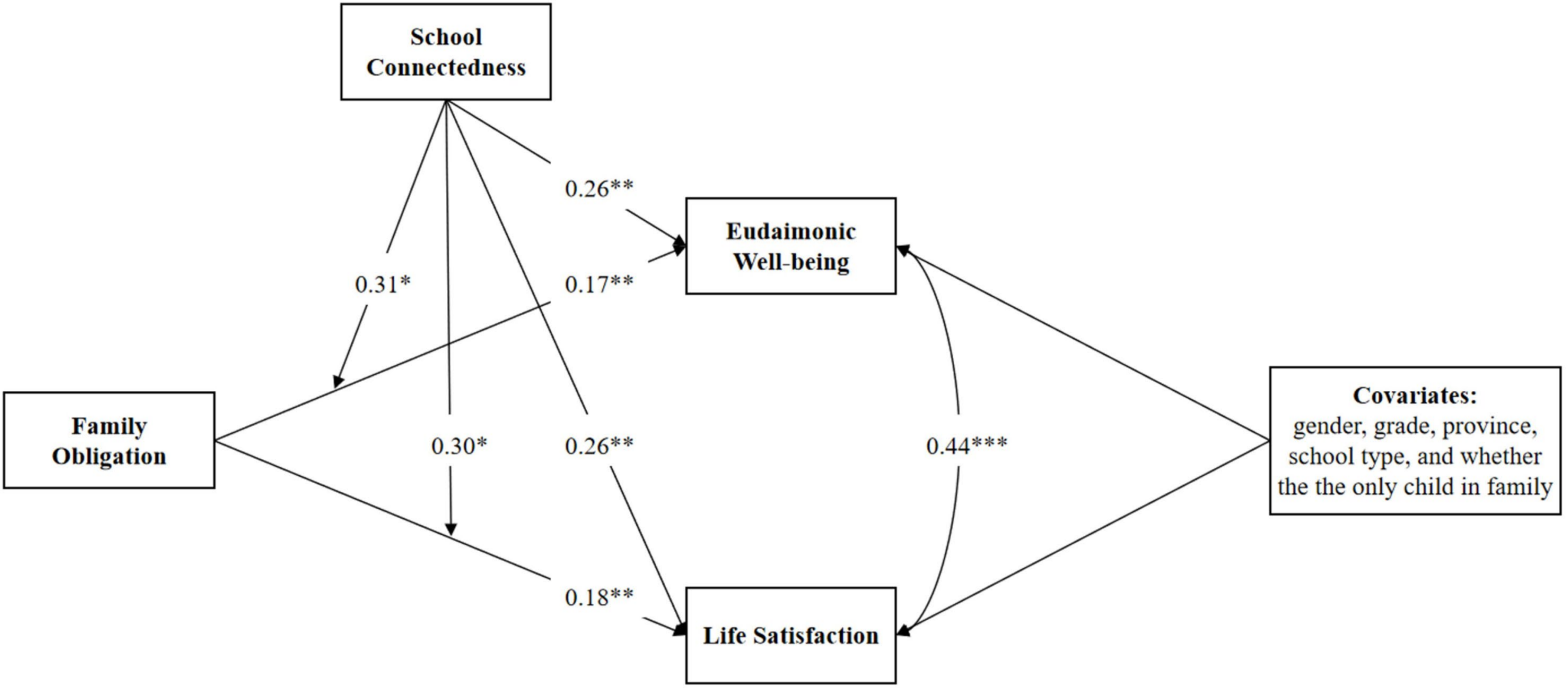
The moderation model linking family obligation, school connectedness, and wellbeing. ^*^p < 0.05; ^**^p < 0.01; ^***^p < 0.001. All coefficients were standardized. All variables were observed variables (uncentered and unstandardized) in the model.

A moderation model was estimated to examine whether school connectedness moderated the association between family obligation and wellbeing (see Figure 2 and Table 4). The model was also just-identified (*df* = 0, CFI = 1.00, RMSEA = 0.00, SRMR = 0.00). After controlling for demographic variables, both family obligation and school connectedness were significantly and positively associated with eudaimonic wellbeing (*β* = 0.17, *p* = 0.003; *β* = 0.26, *p* = 0.002, respectively) and life satisfaction (*β* = 0.18, *p* = 0.002; *β* = 0.26, *p* = 0.001, respectively). Furthermore, the interaction between family obligation and school connectedness was significantly associated with both eudaimonic wellbeing (*β* = 0.31, *p* = 0.011) and life satisfaction (*β* = 0.30, *p* = 0.010).

**Table 4:** Standardized results of the moderating role of school connectedness.

Predictors	Dependent variable							
	Eudaimonic wellbeing (*R*^2^ = 0.49)				Life satisfaction (*R*^2^ = 0.51)			
	*β*	SE	*p*	95% CI	*β*	SE	*p*	95% CI
1. Gender	−0.08	0.02	<0.001	[−0.11, −0.05]	−0.10	0.02	<0.001	[−0.13, −0.07]
2. Grade	0.02	0.02	0.287	[−0.02, 0.05]	−0.03	0.02	0.049	[−0.07, 0.00]
3. Province	−0.05	0.02	0.003	[−0.08, −0.02]	−0.03	0.02	0.034	[−0.07, −0.00]
4. School type	0.01	0.02	0.755	[−0.03, 0.04]	−0.02	0.02	0.166	[−0.06, 0.01]
5. Only child	−0.03	0.02	0.029	[−0.06, −0.00]	−0.05	0.02	0.003	[−0.07, −0.02]
6. Family obligation	0.17	0.06	0.003	[0.06, 0.29]	0.18	0.06	0.002	[0.07, 0.29]
7. School connectedness	0.26	0.08	0.002	[0.10, 0.41]	0.26	0.08	0.001	[0.10, 0.41]
8. Family obligation × school connectedness	0.31	0.12	0.011	[0.07, 0.54]	0.30	0.12	0.010	[0.07, 0.54]

“Only child” refers to “whether the only child in family.” SE, standard error; CI, confidence interval.

**Figure 3:**
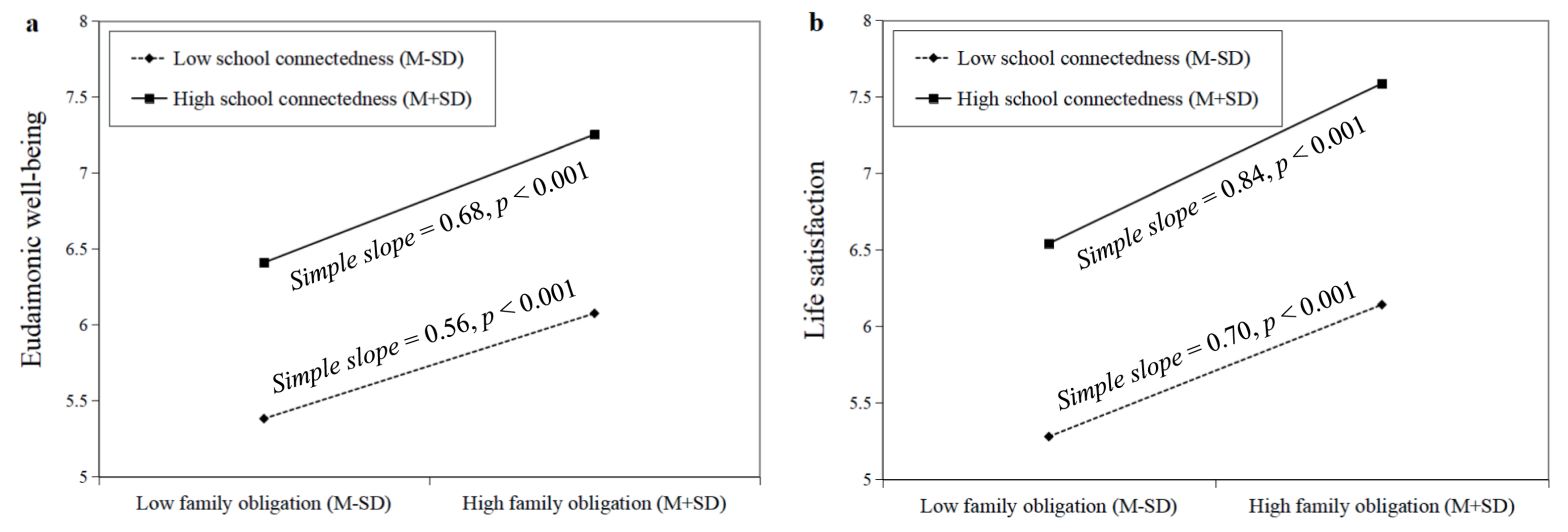
Simple slope analyses for the interaction effects. All coefficients were unstandardized. **(a,b)** Correspond to interaction effects for eudaimonic wellbeing and life satisfaction.

Simple slope analyses based on the unstandardized regression coefficients were conducted to further clarify the nature of these interaction effects (see Figure 3). For eudaimonic wellbeing, the positive association with family obligation was weaker when school connectedness was low (i.e., *M* − 1 SD; *b* = 0.56, *p* < 0.001, 95% CI = [0.49, 0.63]) and stronger when school connectedness was high (i.e., *M* + 1 SD; *b* = 0.68, *p* < 0.001, 95% CI = [0.59, 0.77]). A similar pattern was observed for life satisfaction. Specifically, the positive association between family obligation and life satisfaction was weaker at low levels of school connectedness (*b* = 0.70, *p* < 0.001, 95% CI = [0.60, 0.79]) and stronger at high levels of school connectedness (*b* = 0.84, *p* < 0.001, 95% CI = [0.73, 0.95]).

## 4 Discussion

The present study examined the associations between family obligation and Chinese adolescents’ eudaimonic wellbeing and life satisfaction, as well as the moderating role of school connectedness, using a sample of 2,259 Chinese secondary school students. The results showed that family obligation was positively associated with both eudaimonic wellbeing and life satisfaction. Moreover, school connectedness moderated these associations, such that the positive associations between family obligation and wellbeing were stronger among adolescents with higher levels of school connectedness.

### 4.1 Family obligation was positively associated with adolescents’ wellbeing

Consistent with our hypotheses (H1 and H2), the results revealed that family obligation was positively associated with both eudaimonic wellbeing and life satisfaction among Chinese adolescents, which aligns with the findings of previous studies (Fuligni et al., 2002; Yang et al., 2024). This finding can be interpreted from the perspective of Chinese collectivistic cultural values and the dual-dimensional nature of wellbeing.

In terms of eudaimonic wellbeing, which emphasizes personal growth, purpose, and role fulfillment (Ryan and Deci, 2001), family obligation may provide Chinese adolescents with opportunities to fulfill their family roles, which may help adolescents develop their sense of self-worth, competence, and life meaning. Rooted in filial piety and intergenerational reciprocity, family obligation encourages adolescents to engage in family care, respect their parents, and commit to future family support (Yang et al., 2024). These behaviors may not only align with cultural norms but also help adolescents develop a strong sense of responsibility and social connection, which are core components of eudaimonic wellbeing. This is consistent with Fuligni et al. (2002)’s finding that family obligation is associated with greater psychological wellbeing among Chinese American adolescents, highlighting the cross-cultural consistency of the positive link between family obligation and eudaimonic wellbeing.

Regarding life satisfaction, family obligation may foster positive family interactions and emotional support, which may, in turn, improve life satisfaction for adolescents. Fulfilling family obligations may often elicit positive feedback from family members, such as recognition and affection, which may enhance adolescents’ feelings of joy and contentment (Telzer and Fuligni, 2009). Additionally, the sense of security and belonging derived from a strong family bond—facilitated by family obligation—may reduce negative emotions and improve overall life satisfaction (Yan et al., 2022). Taken together, these findings suggest that family obligation may be associated with both adolescents’ “doing well” (eudaimonic wellbeing) and “feeling good” (life satisfaction), highlighting its potential relevance for multiple aspects of positive psychological functioning.

Although the two dimensions of wellbeing are theoretically distinguishable, the present study revealed highly similar patterns of association with family obligation. This finding suggests that, within the Chinese collectivistic cultural context, family obligation may be associated with both adolescents’ “feeling good” and “functioning well.” Rather than exerting domain-specific influences, family obligation appears to operate as a broad developmental resource that benefits multiple aspects of positive psychological functioning. One possible explanation is that fulfilling culturally valued family responsibilities not only provides adolescents with meaning, competence, and social contribution (thereby promoting eudaimonic wellbeing), but also enhances family harmony, emotional security, and life satisfaction. Consequently, although eudaimonic wellbeing and life satisfaction remain conceptually distinct, they may be jointly associated with the same family processes in collectivistic cultures.

### 4.2 School connectedness moderated the associations between family obligation and adolescents’ wellbeing

The present study further found that school connectedness significantly moderated the associations between family obligation and both dimensions of adolescents’ wellbeing, supporting our hypotheses (H3 and H4) and extending previous findings on the joint role of family and school contexts in adolescent development (Allen et al., 2018; Wilkinson-Lee et al., 2011). Specifically, the positive associations between family obligation and both eudaimonic wellbeing and life satisfaction were stronger among adolescents reporting higher levels of school connectedness.

These findings can be interpreted from the perspective of Bronfenbrenner’s ecological systems theory, which emphasizes that adolescent development is embedded within multiple interacting microsystems rather than being determined by isolated environmental influences (Bronfenbrenner, 1992). From this perspective, family obligation originates within the family microsystem, whereas school connectedness reflects adolescents’ experiences within the school microsystem. The developmental significance of family obligation may therefore depend, at least in part, on adolescents’ experiences in school. Rather than functioning merely as another protective factor associated with wellbeing, school connectedness may provide a broader social context in which the positive meanings of family obligation are reinforced and integrated into adolescents’ daily experiences.

One possible explanation is that adolescents with high levels of school connectedness experience stronger feelings of belonging, more supportive teacher-student relationships, and more positive peer interactions (McNeely et al., 2002; Peng and Patterson, 2025). Such supportive school experiences may reinforce the values underlying family obligation, including responsibility, respect, and care for others. For example, adolescents who assume responsibilities within their families may also receive recognition, encouragement, and social affirmation from teachers and peers. These consistent experiences across family and school contexts may strengthen adolescents’ sense of competence, social value, and belonging, thereby helping explain why family obligation was more strongly associated with both eudaimonic wellbeing and life satisfaction among adolescents with higher school connectedness.

By contrast, adolescents with lower school connectedness may have fewer opportunities to receive emotional support and social affirmation outside the family. Consequently, although they may still report strong family obligation, these family experiences may be less consistently reinforced across different developmental contexts. Under such circumstances, the positive associations between family obligation and wellbeing may be comparatively weaker because the supportive resources available within the school environment are more limited. Consistent with this interpretation, the simple slope analyses showed that the positive associations between family obligation and both dimensions of wellbeing were stronger among adolescents with higher levels of school connectedness than among those with lower levels of school connectedness.

Taken together, these findings extend previous research by suggesting that school connectedness may represent a contextual boundary condition under which family obligation is more strongly associated with adolescent wellbeing. Rather than operating independently, family and school appear to function as interconnected developmental contexts, highlighting the importance of considering cross-contextual processes when examining adolescents’ positive psychological functioning.

### 4.3 Theoretical and practical implications

The present study contributes to the literature in several important ways. First, it extends research on family obligation and adolescent wellbeing by focusing on Chinese adolescents, thereby addressing the relative lack of empirical evidence from non-Western cultural contexts. Whereas previous studies have primarily examined Western or mixed Asian samples (Fuligni et al., 2002; Yan et al., 2022), the present findings suggest that family obligation is positively associated with both eudaimonic wellbeing and life satisfaction among Chinese adolescents. These findings broaden the cultural context in which associations between family obligation and wellbeing have been documented and highlight the importance of considering collectivistic cultural values when examining adolescent development.

Second, the study extends the application of Bronfenbrenner’s ecological systems theory (Bronfenbrenner, 1992) by conceptualizing school connectedness as a contextual boundary condition rather than merely another protective factor associated with wellbeing. Specifically, the findings suggest that the positive associations between family obligation and adolescent wellbeing are stronger among adolescents reporting higher levels of school connectedness. This finding contributes to a better understanding of how family and school microsystems may jointly relate to adolescents’ positive psychological functioning.

Third, by simultaneously examining eudaimonic wellbeing and life satisfaction, the present study provides a more comprehensive understanding of the associations between family obligation and different dimensions of positive functioning. Family obligation showed positive associations with both dimensions of wellbeing, school connectedness moderated these associations. These findings suggest that family obligation may be broadly associated with adolescents’ positive functioning across multiple domains.

The present findings may also have practical implications, although they should be interpreted with appropriate caution given the cross-sectional nature of the study.

From the perspective of family education, the findings suggest that cultivating an appropriate sense of family obligation may be beneficial for adolescents’ positive development. Rather than emphasizing family responsibilities as external demands, parents may consider encouraging adolescents to participate voluntarily in family responsibilities in ways that are developmentally appropriate. Such family experiences may help adolescents develop responsibility, strengthen family relationships, and experience greater meaning and belonging within the family.

From the perspective of school education, the findings highlight the potential importance of fostering school connectedness. Schools may benefit from creating supportive environments characterized by positive teacher-student relationships, constructive peer interactions, and opportunities for meaningful participation in school life (Peng and Patterson, 2025). Although the present findings do not permit causal conclusions, they suggest that supportive school environments may provide conditions under which family-related developmental resources are more strongly associated with adolescents’ positive psychological functioning.

Finally, the present findings may also inform future family- and school-based mental health promotion programs. Rather than focusing exclusively on either the family or the school context, future intervention studies may consider integrating both contexts when designing programs to support adolescent wellbeing. Longitudinal and intervention research will be needed to determine whether strengthening family obligation, school connectedness, or both can lead to improvements in adolescents’ wellbeing.

### 4.4 Limitations and future directions

Despite its contributions, the present study has several limitations. First, although life satisfaction represents the cognitive component of hedonic wellbeing and is widely used as an indicator of subjective wellbeing (Busseri and Sadava, 2011; Diener et al., 2018), the present study did not assess the affective components of hedonic wellbeing (i.e., positive and negative affect). Therefore, the findings should not be generalized to the broader construct of hedonic wellbeing. Future research could incorporate both cognitive and affective indicators to provide a more comprehensive assessment of hedonic wellbeing and examine whether the observed associations generalize across different facets of wellbeing. Second, the study adopted a cross-sectional design, which precludes causal inferences regarding the associations among family obligation, school connectedness, eudaimonic wellbeing, and life satisfaction. Future longitudinal or experimental studies are needed to examine the temporal ordering of these variables and to better understand their developmental relationships.

Third, the current sample was recruited through convenience sampling from three Chinese provinces with uneven provincial and urban–rural distributions. In addition, this study did not systematically collect detailed school-level information, such as school identifiers, the total number of participating schools, school administrative characteristics, or school ranking. Consequently, we were unable to examine whether participants were clustered within schools or account for potential school-level non-independence in the analyses. Although the present study focused on individual-level psychological associations, ignoring possible clustering effects may have influenced the precision of parameter estimates. Furthermore, the lack of detailed school-level information limits the assessment of sample representativeness. Future studies should adopt stratified sampling procedures, systematically collect school-level information, and apply multilevel analytical approaches when appropriate to account for the nested structure of educational data.

Fourth, the study relied solely on self-report measures (Zhang et al., 2025), which may introduce potential biases (e.g., social desirability bias). Future research could adopt a multi-source measurement approach, incorporating reports from parents, teachers, and peers, to obtain more objective data. Finally, future research could explore potential cultural differences in the relationship between family obligation, school connectedness, and wellbeing by comparing Chinese adolescents with adolescents from other cultural backgrounds. This would help to further enhance the cross-cultural understanding of adolescent wellbeing development.

## 5 Conclusion

The present study showed that family obligation was positively associated with both eudaimonic wellbeing and life satisfaction among Chinese adolescents. In addition, school connectedness moderated these associations, such that the positive associations between family obligation and both dimensions of wellbeing were stronger among adolescents with higher levels of school connectedness. These findings extend the application of ecological systems theory by highlighting the importance of interactions between family and school contexts in understanding adolescent wellbeing. Although causal conclusions cannot be drawn from the present cross-sectional study, the findings provide a foundation for future longitudinal and intervention research examining how family and school experiences jointly relate to adolescents’ positive psychological functioning.

## Data Availability

The raw data supporting the conclusions of this article will be made available by the authors, without undue reservation.

## Footnotes

1 Wenjuanxing: http://www.wjx.cn/.
